# Supplementary material for: Spatial Distribution, Potential Sources, and Health Risk of Polycyclic Aromatic Hydrocarbons (PAHs) in the Surface Soils under Different Land-Use Covers of Shanxi Province, North China
Source: Int J Environ Res Public Health. 2022 Sep 21;19(19):11949. doi: 10.3390/ijerph191911949 (PMC9565183; doi:10.3390/ijerph191911949)
Supplement: Supplementary file 1 [file ijerph-19-11949-s001.zip › ijerph-1878341-supplementary.pdf]

SUPPLEMENTARY INFORMATION

Health risk assessment method

BaP toxic equivalent concentration (BaP$_{eq}$), a common evaluation method, was applied to assess the health risk of PAHs in many studies (Table S1).

$$BaP_{eq} = \sum_{i}^{n} C_i \times TEF_i \quad (1)$$

where C_i is the concentration of ith PAH, n is the number of individual PAH, TEF_i is the corresponding toxicity equivalent factor (TEF) of ith PAH.

Table S1. Toxic equivalent concentration (BaP_{eq}) of PAHs in soil of Shanxi Province.

PAHs	Limit value（ng/g）		BaP_{eq}（ng/g）		
		TEF_i	Minimum	Maximum	Mean
Nap	15	0.001	0.00	0.09	0.02
Acy		0.001	0.00	0.05	0.00
Ace		0.001	0.00	0.17	0.00
Flu		0.001	0.00	0.05	0.01
Phe	50	0.001	0.01	0.52	0.04
Ant	50	0.01	0.00	0.84	0.05
Fla	15	0.001	0.00	1.11	0.04
Pyr		0.001	0.00	0.83	0.03
Baa[c]	20	0.1	0.12	60.64	1.89
Chr[c]	20	0.01	0.02	5.31	0.26

Bbf[c]		0.1	0.23	95.49	3.89
Bkf[c]	25	0.1	0.10	42.80	1.44
Bap[c]	25	1	0.62	549.54	15.74
Icdp[c]	25	0.1	0.09	42.09	1.51
Daa[c]		1	0.18	171.22	5.70
Bgp	20	0.01	0.01	4.38	0.19
∑7[c]PAHs			0.96	180.40	20.57
∑16PAHs			1.02	184.00	21.09

[c] Carcinogenic PAHs.

The carcinogenic risk (CR) of soil PAHs was calculated by three pathways including accidental ingestion, skin contact, and respiratory ingestion. The cancer risk calculation formula of these three ways is as equation (2)-(5):

$$CR_{ingestion} = \frac{C_{PAHs} \times IR_{soil} \times EF \times ED \times CF}{BW \times AT} \times CSF_{ingestion} \quad (2)$$

$$CR_{skin} = \frac{C_{PAHs} \times SA \times AF_{PAHs} \times ABS \times EF \times ED \times CF}{BW \times AT} \times CSF_{skin} \quad (3)$$

$$CR_{respiratory} = \frac{C_{PAHs} \times IR_{air} \times EF \times ED \times CF}{PEF \times BW \times AT} \times CSF_{respiratory} \quad (3)$$

$$CR = CR_{ingestion} + CR_{skin} + CR_{respiratory} \quad (5)$$

Where C_{PAHs} is the concentration of soil PAHs, *IR* is soil and air uptake rate, *EF* is exposure frequency, *ED* is exposure duration, *CF* is transformation factor (10^{-6} kg mg^{-1}), *BW* is body weight, *AT* is average life span, *CSF* is the slope factor of accidental ingestion of cancer ($CSF_{ingestion}$ = 7.3 kg D^{-1} mg^{-1}, CSF_{skin} = 25 kg D^{-1} mg^{-1}, $CSF_{respiratory}$ = 3.9 kg D^{-1} mg^{-1}), *AF* is skin adhesion factor, *ABS* is adsorption factor, *PEF* is soil particle emission factor. Parameters used in the cancer risk assessment is listed in Table S2.

Table S2. Parameters used in the cancer risk assessment.

Parameters	Male child	Male youth	Male adult
BW (kg)	17.2	47.1	62.8
EF (d year^{-1})	350	350	350
ED (year)	6	14	30
IR_{air} (m^3 d^{-1})	7.6	12.9	13.1
IR_{soil} (mg d^{-1})	100	100	50
SA (cm^2)	1090	1900	2120
AF_{soil} (mg cm^{-2})	0.2	0.2	0.07
ABS	0.13	0.13	0.13
AT (d)	25550	25550	25550
PEF (m^3 mg^{-1})	1.4×10^9	1.4×10^9	1.4×10^9

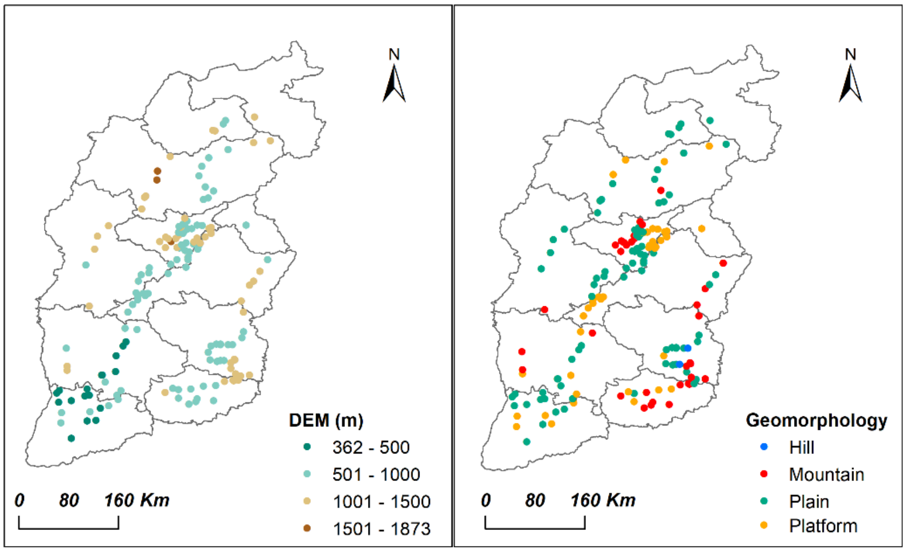

Figure S1. Altitude and geomorphology information of sampling sites.
